# The construction and analysis of tumor-infiltrating immune cell and ceRNA networks in recurrent soft tissue sarcoma

**DOI:** 10.18632/aging.102424

**Published:** 2019-11-18

**Authors:** Runzhi Huang, Tong Meng, Rui Chen, Penghui Yan, Jie Zhang, Peng Hu, Xiaolong Zhu, Huabin Yin, Dianwen Song, Zongqiang Huang

**Affiliations:** 1 Department of Orthopaedics, The First Affiliated Hospital of Zhengzhou University, Zhengzhou 450052, China; 2 Division of Spine, Department of Orthopedics, Tongji Hospital Affiliated to Tongji University School of Medicine, Shanghai 200065, China; 3 Department of Orthopedics, Shanghai General Hospital, School of Medicine, Shanghai Jiaotong University, Shanghai 200080, China; 4 Department of Urology, Shanghai Changhai Hospital, Second Military Medical University, Shanghai 200433, China; 5 Shanghai East Hospital, Key Laboratory of Arrhythmias, Ministry of Education, Tongji University School of Medicine, Shanghai 200120, China

**Keywords:** soft tissue sarcoma, bone tumor, recurrence, ceRNA, immune cell, prognosis

## Abstract

Soft tissue sarcoma (STS) is one of the most challenging tumors for medical oncologists, with a high rate of recurrence after initial resection. In this study, a recurrent STS-specific competitive endogenous RNA (ceRNA) network including seven recurrence and overall survival (OS)-associated genes (LPP-AS2, MUC1, GAB2, hsa-let-7i-5p, hsa-let-7f-5p, hsa-miR-101-3p and hsa-miR-1226-3p) was established based on the gene expression profiling of 259 primary sarcomas and 3 local recurrence samples from the TCGA database. The algorithm “cell type identification by estimating relative subsets of RNA transcripts (CIBERSORT)” was applied to estimate the fraction of immune cells in sarcomas. Based on 5 recurrence and OS-associated immune cells (NK cells activated, dendritic cells resting, mast cells resting, mast cells activated and macrophages M1), we constructed a recurrent STS-specific immune cells network. Both nomograms were identified to have good reliabilities (Area Under Curve (AUC) of 5-year survival is 0.724 and 0.773, respectively). Then the co-expression analysis was performed to identify the potential regulation network among recurrent STS-specific immune cells and ceRNAs. Hsa-miR-1226-3p and MUC1 were significantly correlated and dendritic cells resting was related to hsa-miR-1226-3p. Additionally, the expression of MUC1 and dendritic cell marker CD11c were also verified by immunohistochemistry (IHC) assay and multidimensional databases. In conclusion, this study illustrated the potential mechanism of hsa-miR-1226-3p regulating MUC1 and dendritic cells resting might play an important role in STS recurrence. These findings might provide potential prognostic biomarkers and therapeutic targets for recurrent STS.

## INTRODUCTION

Soft tissue sarcoma (STS) is a group of rare tumors including more than 50 different histological subtypes [1]. It accounts for approximately 1% of adult malignancies and 15 % of pediatric malignancies [2, 3]. STS is derived from mesenchymal cell and usually divided into two broad categories: sarcomas of the soft tissues and sarcomas of the bone [2]. The extremities, viscera, retroperitoneum and trunk are the most frequent sites, accounting for 70% of all cases [3, 4]. A complete resection is recommended for STS, but anatomic constraints hinder such efforts and local recurrence rate is high [5, 6]. Even after radical surgeries, about 30% of patients would experience local recurrence (LR) within 10 years, which is the most common cause of death [7]. Thus, there is a pressing need to explore the underlying mechanism of STS recurrence, which may provide potential prognostic factors and therapeutic targets for its treatment in the clinic.

Both tumor cells and tumor-infiltrating immune cells participate in tumorigenesis and tumor progression [9] and have been confirmed to be associated with recurrence and overall survival (OS) [10, 11]. The crosstalk between the tumor cells and tumor-infiltrating immune cells is usually modulated by the competing endogenous RNA (ceRNA) networks, which are composed of mRNAs messenger RNAs (mRNAs), long non-coding RNAs (lncRNAs), and microRNAs (miRNAs) [12]. Increasing studies indicated that the ceRNA networks regulate the post-transcription of oncogenes and tumor suppressor genes, modulate interactions between protein and genes, and control the biological behaviors such as tumor invasion and metastasis [12]. However, no combined networks have been defined for predicting the prognosis of STS recurrence up to date. Therefore, a better understanding of the tumor-infiltrating immune cells and ceRNA networks is required.

In the current study, we identified the differential expressed ceRNAs involved in recurrent STSs based on their gene expression profiling available from the TCGA (The Cancer Genome Atlas) database and used the algorithm “CIBERSORT” to quantify the proportions of immune cells. In addition, prediction nomograms based on recurrence and OS-associated immune cells or ceRNAs were constructed to predict STS recurrence. Moreover, we assessed the relationships between recurrent STS-specific immune cells and ceRNA networks to identify the underlying immune gene signature.

## RESULTS

### Identification of significantly differentially expressed genes

**Figure 1:**
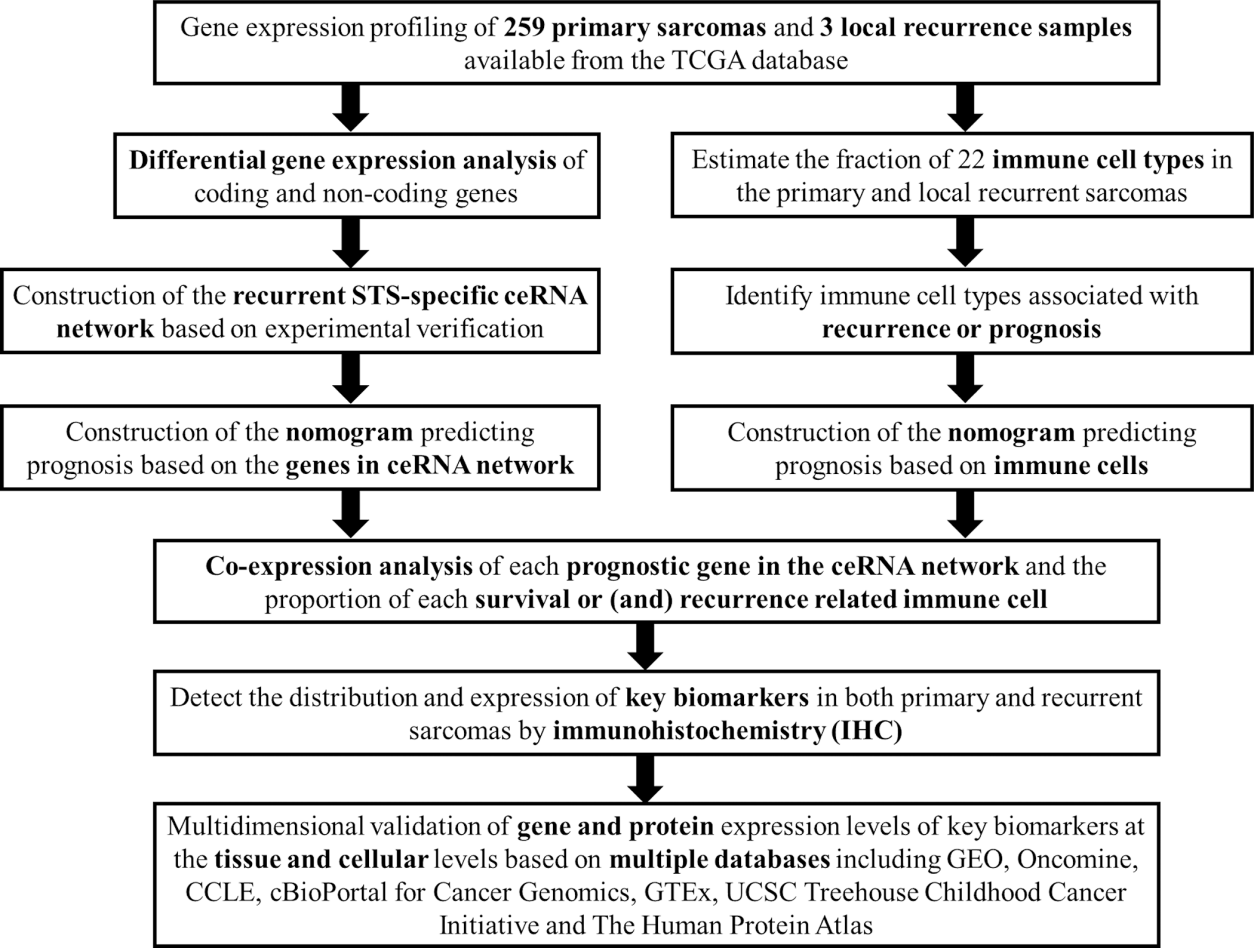
**The flow chart of the analysis process.** Abbreviations: TCGA: The Cancer Genome Atlas; STS: Soft tissue sarcoma; GEO: Gene Expression Omnibus; CCLE: Cancer Cell Line Encyclopedia; GTEx: Genotype-Tissue Expression; UCSC: University of California, Santa Cruz.

Figure 1 summarizes the analysis process of this study. The baseline characteristics of all the patients available from the TCGA database are listed in Supplementary Table 1. The Kaplan-Meier survival analysis revealed that recurrence was a significant predictor for poor prognosis of STSs (P = 0.001) (Supplementary Figure 1).

**Figure 2:**
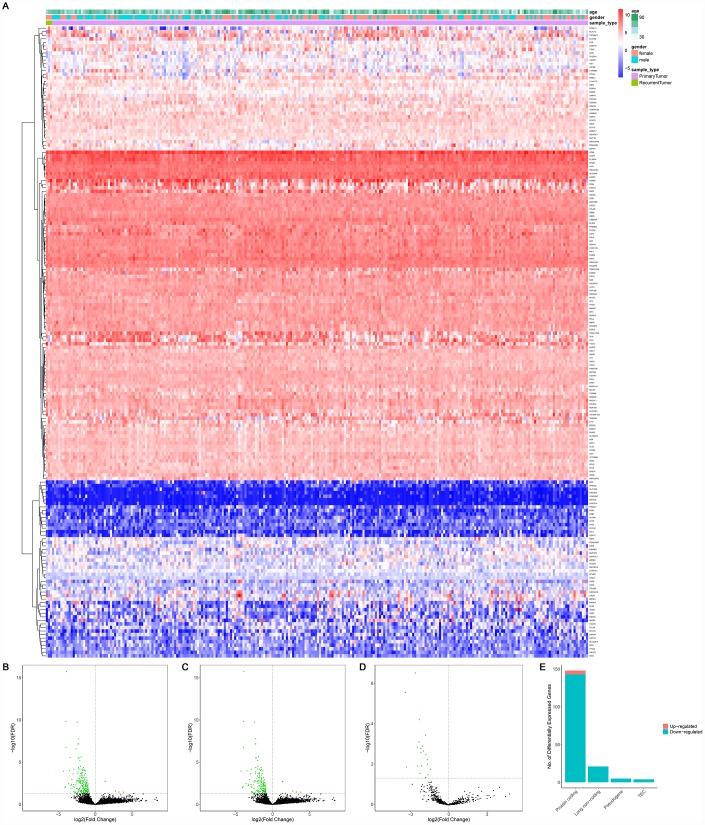
**The differentially expressed genes between primary and recurrent STSs.** (**A**) The heatmap and the volcano plot (**B**) of 178 differentially expressed genes between 259 primary and 3 recurrent STSs; (**C**) The volcano plot of 148 differentially expressed protein-coding genes between 259 primary and 3 recurrent STSs; The volcano Plot (**D**) of 21 differentially lncRNAs between 259 primary and 3 recurrent STSs; (**E**) The composition of differentially expressed genes. The log(fold-change) > 1.0 or < -1.0 and FDR < 0.05. Abbreviations: ceRNAs: competing endogenous RNAs; STSs: soft tissue sarcomas; LncRNA: long non-coding RNA.

A total of 14,447 lncRNAs, 2,588 miRNAs and 19,660 mRNAs were found from the TCGA database. Among them, 148 differentially expressed protein-coding genes (143 downregulated and 5 upregulated) (Figure 2A–2C, 2E), 21 differentially expressed lncRNAs (downregulated) (Figure 2A, 2D) and 4 differentially expressed miRNAs (downregulated) were identified between primary and recurrent STSs using the cutoff of the log (fold-change) > 1.0 or < −1.0 and FDR < 0.05. Supplementary Table 2 summarizes the top 10 downregulated and top 10 upregulated genes in differential gene analysis.

### Construction of the ceRNA network and survival analysis

**Figure 3:**
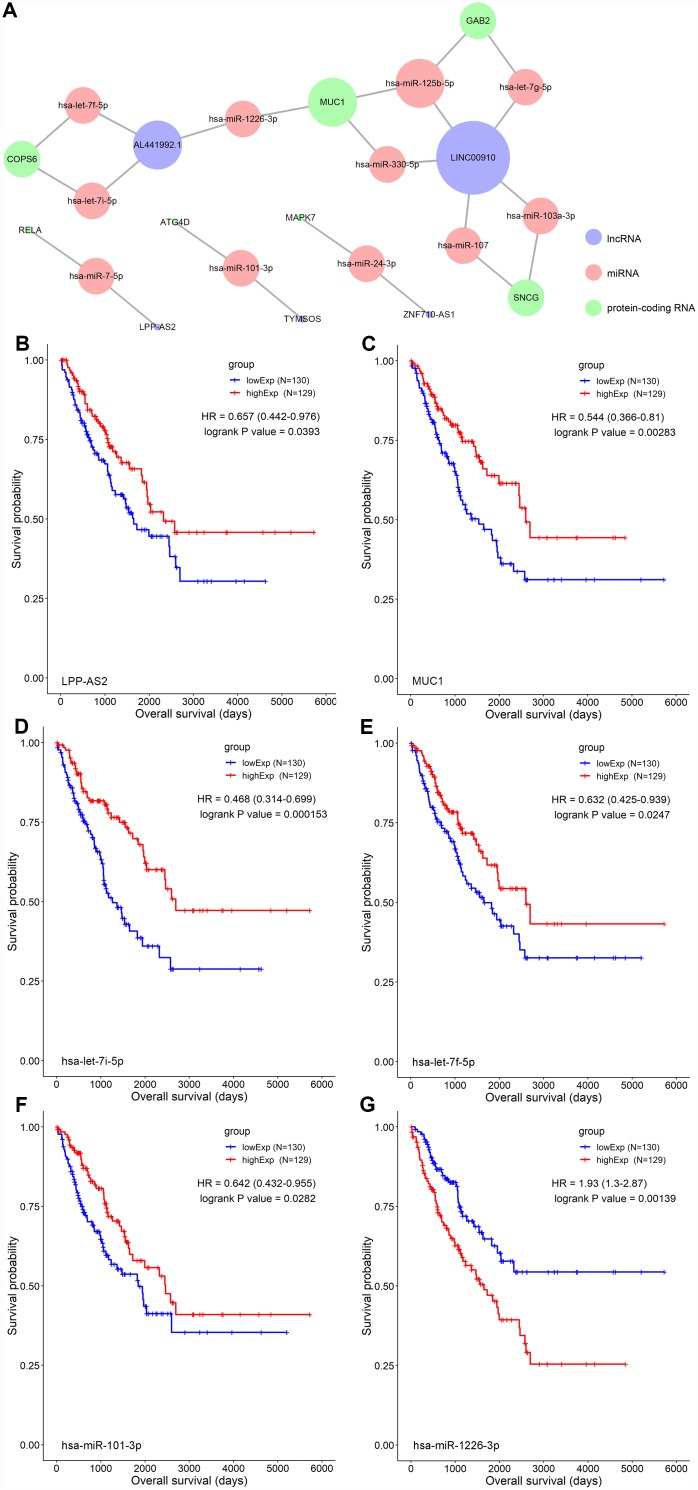
(**A**) The STS-recurrence related ceRNA network; The Kaplan-Meier survival curves of LPP-AS2 (**B**), MUC1 (**C**), hsa-let-7i-5p (**D**), hsa-let-7f-5p (**E**), hsa-miR-101-3p (**F**) and hsa-miR-1226-3p (**G**). Abbreviations: STSs: soft tissue sarcomas; ceRNAs: competing endogenous RNAs

**Figure 4:**
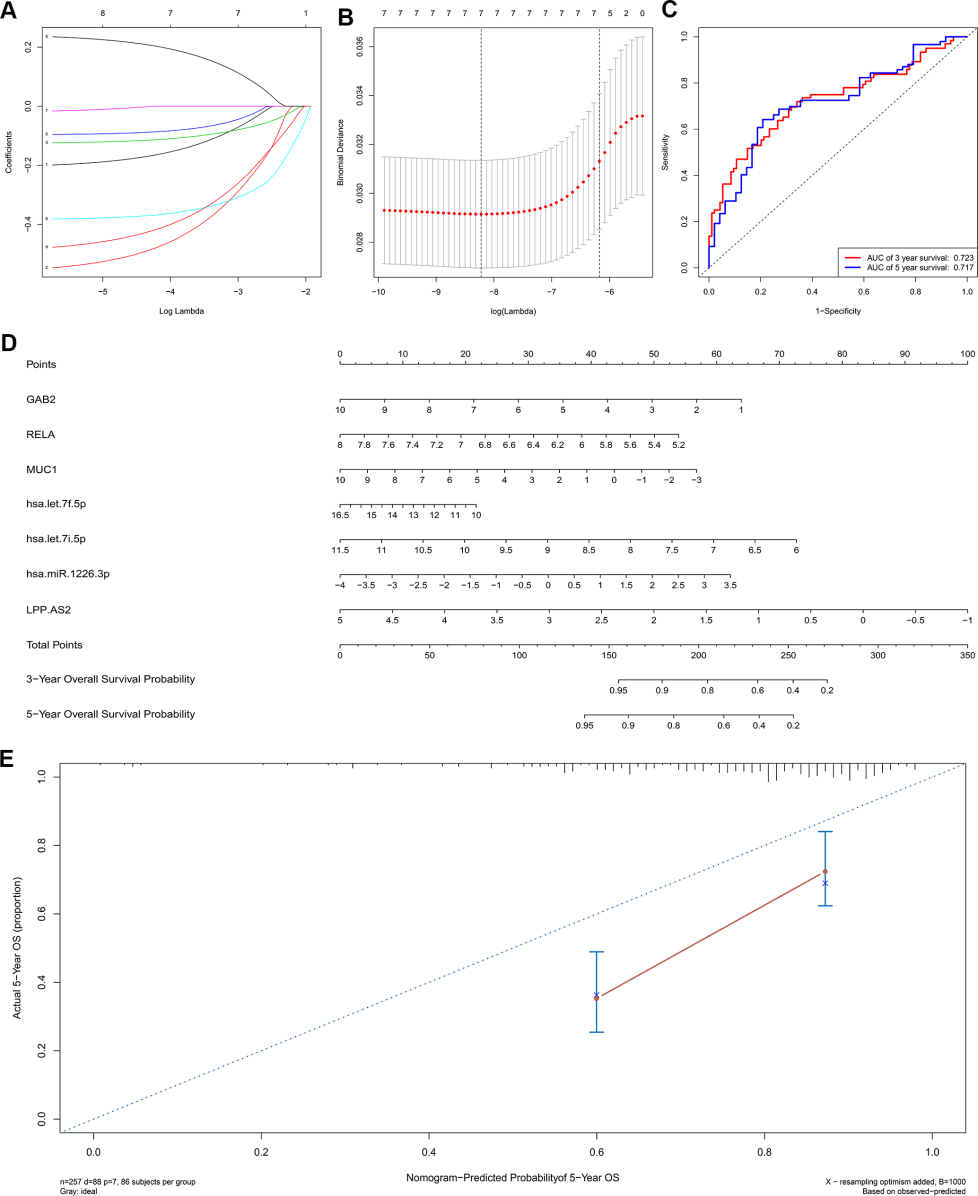
The results of the multivariate Cox regression, nomogram (**E**) and model diagnosis process (**B**, **C**, **D**, **F**) based on the key members in the ceRNA network. Seven potential prognosis-related ceRNAs were integrated into a new multivariable model. The results of the Lasso regression suggested that all seven genes were essential for modeling (**A**, **B**). The nomogram was constructed based on the model (**D**). The ROC and the calibration curves indicated acceptable accuracy (Area Under Curve (AUC) of 3-year survival: 0.731; AUC of 5-year survival: 0.724) and discrimination of the nomogram (**C**, **E**).

A ceRNA network including 23 genes was established based on the interactions of 11 lncRNA-miRNA pairs and 12 miRNA-mRNA pairs (Figure 3A) (Table 1). The Cox regression and Kaplan-Meier method were applied to examine the relationship between the biomarkers in the recurrence-associated ceRNAs and OS. LPP-AS2 (P = 0.039), MUC1 (P = 0.003), GAB2 (P =0.049), hsa-let-7i-5p (P < 0.001), hsa-let-7f-5p (P = 0.025), hsa-miR-101-3p (P = 0.028) and hsa-miR-1226-3p (P = 0.001) were significantly associated with survival in Kaplan-Meier analysis (Figure 3B–3G). Seven potential recurrence and OS-associated biomarkers were identified as key molecules in the ceRNA network and were integrated into a new multivariable model (Table 2). The results of the Lasso regression indicated that all seven genes were essential for modeling (Figure 4A, 4B). Additionally, the ROC and the calibration curves indicated decent accuracy (Area Under Curve (AUC) of 3-year survival: 0.731; AUC of 5-year survival: 0.724) and good discrimination (Figure 4C, 4E). Then, the nomogram was constructed based on the model (Figure 4D).

**Table 1:** Hypergeometric testing and correlation analysis results of ceRNAs network.

**LncRNA**	**Protein-coding RNA**	**MiRNAs**	**Correlation P**	**Hypergeometric test P**
AL441992.1	MUC1	hsa-miR-1226-3p	2.81E-10	0.044178237
AL441992.1	COPS6	hsa-let-7f-5p, hsa-let-7i-5p	8.92E-07	0.000306228
LINC00910	GAB2	hsa-let-7g-5p, hsa-miR-125b-5p	0.002903258	0.005948459
LINC00910	MUC1	hsa-miR-125b-5p, hsa-miR-330-5p	4.29E-07	0.014261321
LINC00910	SNCG	hsa-miR-103a-3p, hsa-miR-107	4.46E-05	0.009708465
TYMSOS	ATG4D	hsa-miR-101-3p	3.68E-05	0.011207883
LPP-AS2	RELA	hsa-miR-7-5p	0.020065721	0.02784492
ZNF710-AS1	MAPK7	hsa-miR-24-3p	0.013123574	0.001872659

Abbreviations: ceRNAs: Competing endogenous RNAs; LncRNA: Long non-coding RNA; MiRNA: microRNA.

* P < 0.05

**Table 2:** Cox proportional hazards regression model including the key members of the ceRNA network for overall survival in patients with soft tissue sarcoma.

**Gene**	**Hazard ratio**	**95%CI**	**P value**
GAB2	0.81	(0.67 − 0.98)	0.034 *
RELA	0.57	(0.33 − 0.98)	0.041 *
MUC1	0.88	(0.79 − 0.97)	0.012 *
has-let-7f-5p	0.91	(0.73 − 1.13)	0.375
has-let-7i-5p	0.68	(0.50 − 0.92)	0.012 *
has-miR-1226-3p	1.28	(1.03 − 1.58)	0.024 *
LPP-AS2	0.61	(0.44 − 0.84)	0.003 **

Abbreviations: ceRNAs: Competing endogenous RNAs; CI: Confidence Interval.

*P < 0.05; **P < 0.010; ***P < 0.001.

Note. In the variable selection process, first of all, the initial Cox models including all members of the ceRNA network were used to select potential prognostic genes. At the same time, the Lasso regression was performed based on all members of the ceRNA network. The results of the Lasso regression (Figure 4A, 4B) suggested that the eight genes were essential to modeling and ensuring not overfitting of the model. Eventually, the reduce Cox model shown in this table only including eight genes filtrating by the Lasso regression (The Cox model of immune cells was constructed in the same way).

### Composition of immune cells in sarcomas

**Figure 5:**
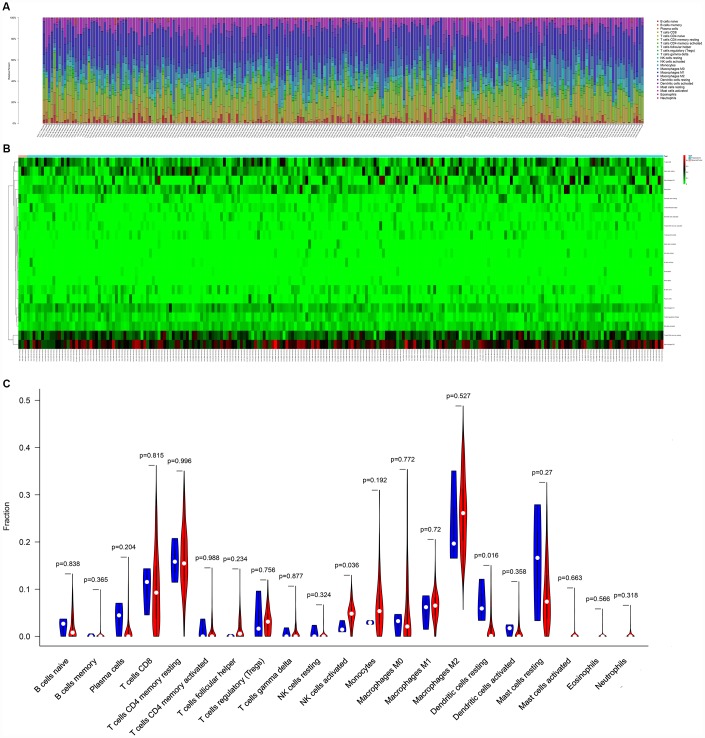
The composition (**A**) and heatmap (**B**) of immune cells estimated by CIBERSORT algorithm in sarcomas. (**C**) The violin plot of immune cells (The blue and red bar stand for recurrent tumor group and primary tumor group, respectively). Abbreviations: CIBERSORT: Cell type identification by estimating relative subsets of RNA transcripts.

Figure 5 illustrated the composition of immune cells estimated by the CIBERSORT algorithm in STSs. The fraction of the NK cells activated was consistently lower in the local recurrence tissue than in primary sarcomas, whereas the fractions of dendritic cells resting and the mast cells resting were higher in the local recurrence sarcoma tissue. Wilcoxon rank-sum test was then used and revealed that the fractions of dendritic cells resting (P = 0.016) and NK cells activated (P = 0.036) varied significantly between recurrent and primary tumors (Figure 5C).

### Integrated analysis of immune cells, genes and prognosis

**Figure 6:**
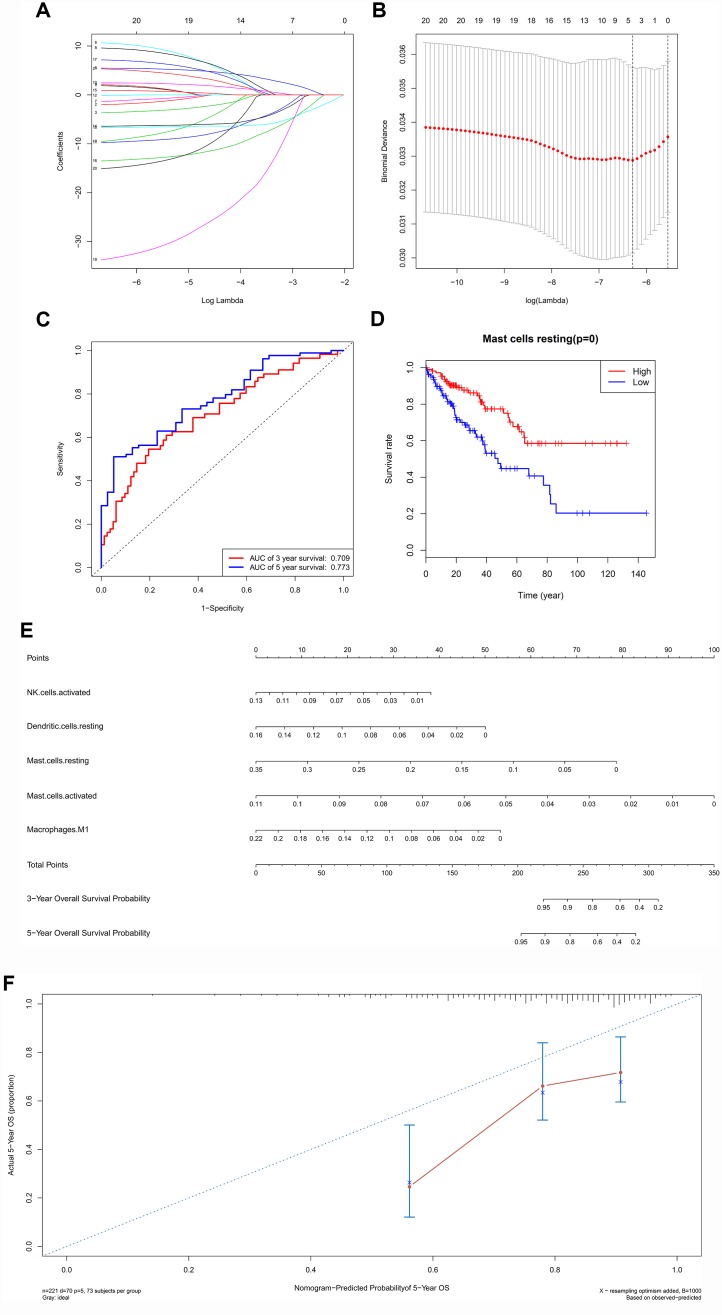
The results of the multivariate Cox regression, Lasso regression (**A**, **B**), Kaplan–Meier survival curve of (**D**), nomogram (**E**) and model diagnosis process (**C**, **F**) based on prognosis related immune cells. All immune cells were integrated into an initial Cox regression model. After the screening process of the Lasso regression, the results suggested that the model was not overfitting (**A**, **B**). The nomogram based on the multivariable model (**E**). The calibration curve and the ROC demonstrated good discrimination and concordance of the nomogram (AUC of 3-year survival: 0.709; AUC of 5-year survival: 0.773) (**C**, **F**).

**Figure 7:**
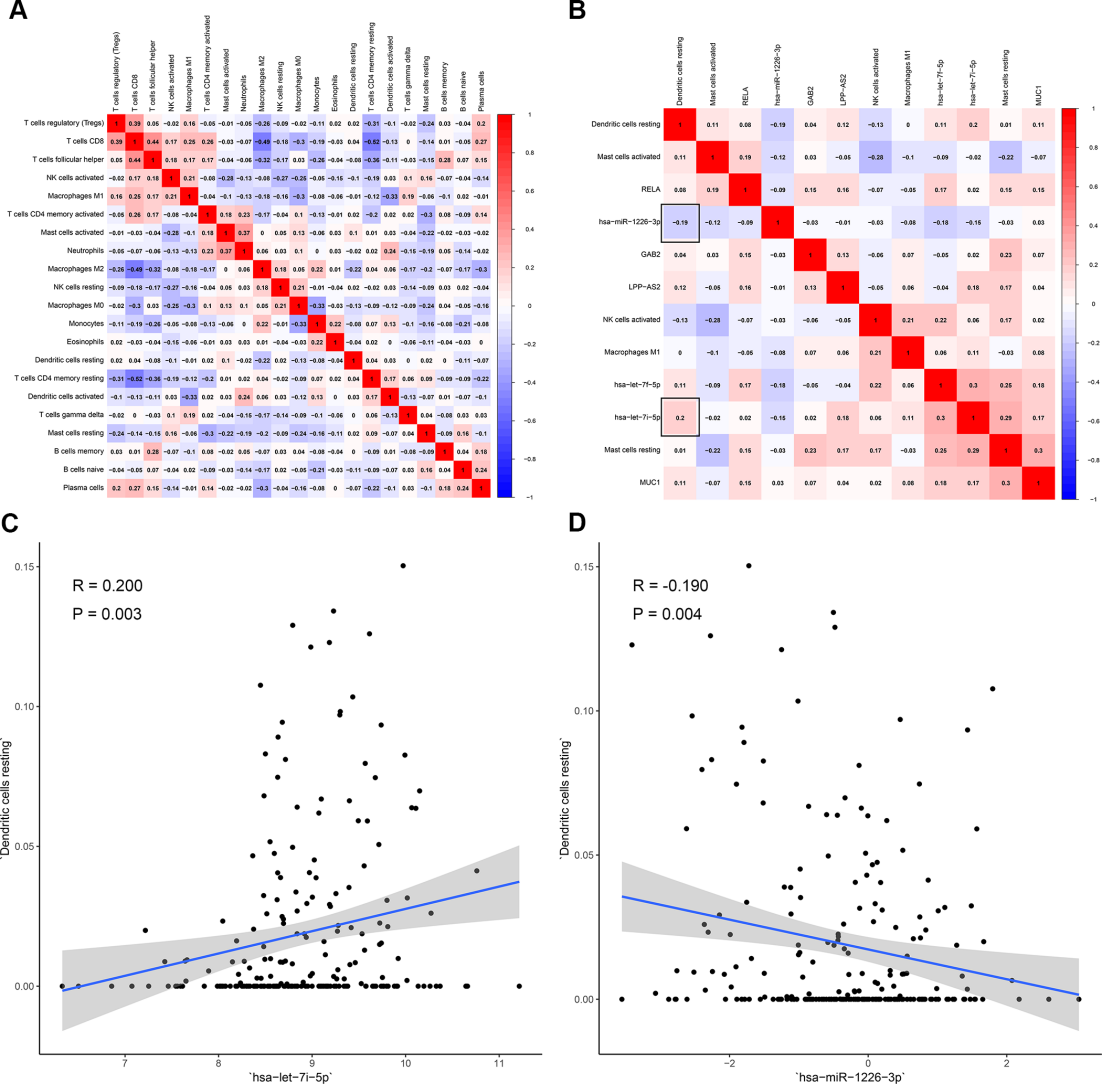
**The co-expression patterns among fractions of immune cells and key members in the ceRNA network.** (**A**) co-expression heatmap of all immune cells; (**B**) co-expression heatmap of prognostic immune cells and key members of ceRNA network; (**C**) has-let-7i-5p was significantly associated with dendritic cells resting (R = 0.200, P = 0.003); (D) hsa-miR-1226-3p was significantly associated with dendritic cells resting (R = -0.190, P = 0.004).

All immune cells were integrated into a Cox regression model. After the screening process of the Lasso regression, the fractions of NK cells activated (P = 0.029), dendritic cells resting (P = 0.013), mast cells resting (P < 0.001), mast cells activated (P = 0.030) and macrophages M1 (P = 0.024) were all considered as independent predictors in the final Cox model (Table 3). The results of the Lasso regression suggested that the model was not overfitting (Figure 6A, 6B). In addition, the calibration curve and the ROC demonstrated good discrimination and concordance (AUC of 3-year survival: 0.709; AUC of 5-year survival: 0.773) (Figure 6C, 6F). Similarly, the nomogram based on the multivariate analysis was constructed (Figure 6E). Lastly, immune cells and biomarkers significantly associated with OS were integrated into the nomogram (Supplementary Figure 2) for predicting the prognosis (AUC of 3-year survival: 0.789; AUC of 5-year survival: 0.822). With respect of the correlation analysis, significant co-expression patterns between fractions of immune cells and key molecules in the ceRNA network were identified, showing that hsa-miR-1226-3p was significantly associated with dendritic cells resting (R= -0.19, P = 0.004) (Figure 7). Additionally, according to the result of the Wilcoxon rank-sum test, hsa-miR-1226-3p was significant different between the sarcoma tissues of patients with and without recurrence (P = 0.015) (Supplementary Figure 3).

**Table 3:** Cox proportional hazards regression model including the key immune cells for overall survival in patients with soft tissue sarcoma.

**Immune cell**	**Hazard ratio**	**95%CI**	**P value**
NK cells activated	9.6e−06	(3.0e−10 − 0.309)	0.029 *
Dendritic cells resting	4.4e−06	(2.7e−10 − 0.074)	0.013 *
Mast cells resting	1.4e−04	(1.9e−06 − 0.011)	< 0.001 ***
Mast cells activated	2.9e−16	(2.4e−30 − 0.034)	0.030 *
Macrophages M1	7.2e−05	(1.8e−08 − 0.280)	0.024 *

Abbreviations: CI: Confidence Interval.

*P < 0.05; **P < 0.010; ***P < 0.001.

### MUC1 and CD11c were associated with STS recurrence

**Figure 8:**
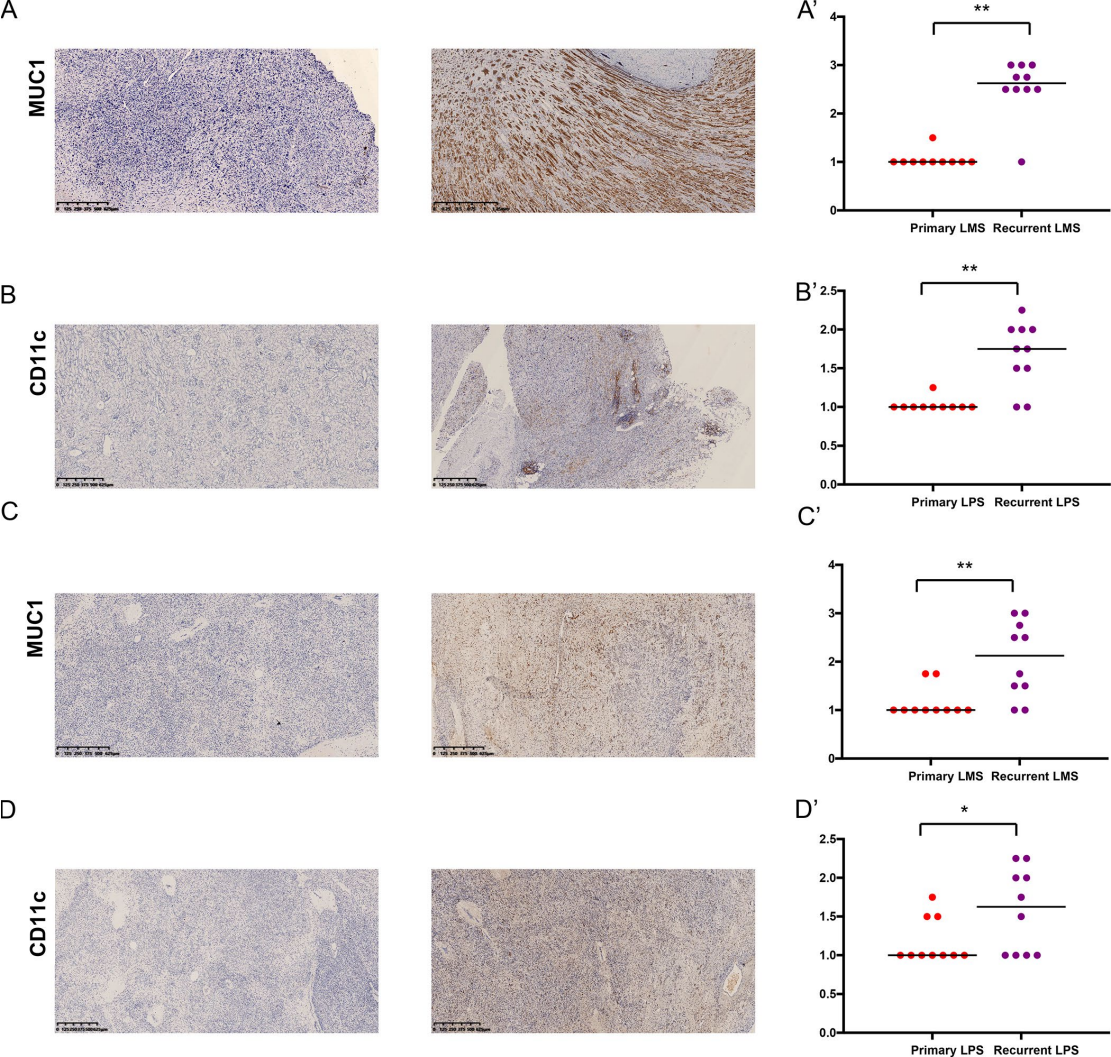
The expressions of MUC1 and CD11c proteins in primary/ recurrent leiomyosarcoma (LMS) (**A**, **B**) and liposarcoma (LPS) (**C**, **D**) specimens examined by immunohistochemistry (IHC) assay. The upper one is primary STS and under one is recurrent STS.

We examined the expressions of MUC1 and CD11c in primary and recurrent leiomyosarcoma (LMS) and liposarcoma (LPS) specimens (Table 4). Of the 10 patients with recurrent LMS, the mean H-score of MUC1 was 2.25, which was significantly higher than that of patients with primary LMS (P < 0.05) (Figure 8A, 8A’). The results of CD11c were similar (Figure 8B, 8B’). Then we compared the H-score of MUC1 and CD11c in patients with primary or recurrent LPS. Both of them were significantly higher in patients with recurrent LPS (Figure 8C, 8C’, 8D, 8D’). In addition, the results showed that the MUC1 and CD11c protein was predominantly localized in the membrane and extracellular matrix of LMS (Supplementary Figure 4A, 4B) and LPS cells (Supplementary Figure 4C, 4D).

**Table 4:** The mean H-score of MUC1 and CD11c in primary/recurrent LMS and LPS.

**Biomarker**	**Primary LMS**	**Recurrent LMS**	**Primary LPS**	**Recurrent LPS**
MUC1	1.05	2.55	1.15	2.05
CD11c	1.025	1.675	1.175	1.625
P value	<0.001	<0.001	0.004	0.42

Abbreviations: LMS: leiomyosarcoma; LPS: liposarcoma.

### Multidimensional validation

A dimensional validation was performed to explore the expressions of MUC1 and CD11c in the primary STS, normal soft tissue and cell lines (Table 5). First, MUC1 (Median rank 1,052, P = 0.012) was highly expressed in primary STS compared to normal tissue while CD11c (Median rank 10,499, P = 0.952) showed no difference in all of the 10 comparisons (Supplementary Figure 5). At cellular level, MUC1 was expressed in various STS cell lines while the expression of CD11c was low (Supplementary Figure 6). Besides, an analysis of genomics and clinical profiles with the cBioPortal suggested that MUC1 and CD11c were highly expressed in primary STS compared to other types of malignancies and both were in a co-expression relationship (R = 0.28, P < 0.001) (Supplementary Figure 7). Moreover, we extracted the RNA-seq data of 907 normal adipose or muscle tissues from the GTEx database and 509 sarcomas from the Treehouse for differential gene analysis. MUC1 (logFC = 4.10, P < 0.001) was identified in the inter-group differential expression while CD11c was not (Supplementary Figure 8). Additionally, the results from The Human Protein Atlas showed that the protein MUC1 and CD11c were almost not detected in normal adipose and smooth muscle tissue (Supplementary Figure 9). Finally, we also evaluated the prognostic value and relationship among MUC1 and twelve markers of dendritic cell. The results revealed that CD49d, CD304, CD209, CD11b and CD86 had a co-expression pattern with MUC1 and CD40, CD197, CD205 were associated with OS (Supplementary Figure 10).

**Table 5:** Summary of multidimensional external validation results base on multiple databases.

**Database**	**MUC1/MUC1**		**ITGAX/CD11c**		**Results**
	**Cancer**	**Normal**	**Cancer**	**Normal**	
Oncomine	↑	↓	-	-	Across ten analysis, MUC1 was highly expressed in primary STS compared to normal tissue while CD11c (Gene symbol: ITGAX) showed no difference (Supplementary Figure 5).
CCLE	↑	NA	-	NA	At the cellular level, MUC1 was expressed in various STS cell lines while ITGAX expression was low STS cell lines (Supplementary Figure 6).
cBioPortal	↑	↓	-	-	MUC1 and ITGAX were highly expressed in primary STS compared to some other types of cancer and they had a significant co-expression pattern in STS (Supplementary Figure 7).
GTEx	NA	↓	NA	↓	MUC1 and ITGAX were lowly expressed in normal adipose tissue and smooth muscle tissue (Supplementary Figure 8).
UCSC Treehouse	↑	↓	-	-	MUC1 was highly expressed in primary STS compared to normal soft tissue while ITGAX did not (Supplementary Figure 8).
The Human Protein Atlas	NA	ND	NA	ND	Protein MUC1 and CD11c were almost not detected in normal adipose tissue and smooth muscle tissue (Supplementary Figure 9).

Note: “↑” was defined as a significantly upregulated gene; “↓” was defined as a significantly downregulated gene; “-” was defined as a a gene with no significant difference in expression; “NA” was defined as “Not available”; “ND” was defined as “Not detected”;

Abbreviations: CCLE: Cancer Cell Line Encyclopedia; GTEx: Genotype-Tissue Expression; UCSC: University of California, Santa Cruz

## DISCUSSION

STSs are one of the most challenging tumors for medical oncologists, with a high rate of relapse after initial resection [5]. During tumorigenesis and recurrence, molecular and cellular components played important roles and were often regarded as potential prognostic factors [13]. The significant genes that are aberrantly expressed in tumor and tumor-infiltrating immune cells attract our interest, however, very few studies of STS focused on them before. In the present study, we found out the significant tumor-infiltrating immune cells and ceRNAs between primary and recurrent STS. Two prediction nomograms with high efficacy were constructed based on these findings and thus both nomograms might assist clinical oncologists in evaluation of prognosis and recurrence. By comparing the correlation between the recurrence-associated ceRNAs and immune cells, we inferred a potential mechanism of STS recurrence and that was hsa-miR-1226-3p regulating MUC1 and dendritic cells resting. Then multidimensional validation of multiple databases confirmed the reliability of our results.

The ceRNA networks link the function of protein-coding mRNAs with ncRNAs, such as miRNAs and lncRNAs [12]. Previous studies revealed that miRNAs were able to bind to the 3’ untranslated region (3’ UTR) of the target mRNAs in a complementary base-pairing manner and take part in post-transcriptional regulation of oncogenes and antioncogenes [17, 18]. The lncRNAs not only regulated interactions between protein and genes, but also modulated transcription by recruiting chromatin-modifying complexes [19, 20]. Emerging evidence demonstrated their potential roles in controlling the biological process including tumorigenesis, invasion and metastasis [20–22].

In this study, hypergeometric testing and correlation analysis results of the ceRNAs network revealed that hsa-miR-1226-3p (miRNA), MUC1 (protein-coding RNA) and AL441992.1 (lncRNA) were significantly correlated. In the meanwhile, the correlation analysis also revealed that hsa-miR-1226-3p was significantly associated with dendritic cells resting (R= -0.190, P = 0.004). Thus, we inferred that the mechanism of hsa-miR-1226-3p regulating MUC1 and dendritic cells resting might play an important role in STS recurrence.

miR-1226 was reported to be involved in tumorigenesis, angiogenesis and drug resistance in breast cancer and non-small cell lung cancer [23, 24]. The correlation between hsa-miR-1226-3p and MUC1 has also been proved by the previous study which revealed that miR-1226 interacted with the MUC1 mRNA 3’ UTR and induced downregulation of MUC1 [25].

Generally, MUC1 is overexpressed at mucosal surfaces and absent in the skin epithelium and mesenchymal cells [26, 27]. Aberrantly glycosylated MUC1 is often overexpressed in most human epithelial cancers, but not reported in mesenchymal cell originated STS. In the tumorigenesis, MUC1 was reported to induce the expression of growth factors such as connective tissue growth factor (CTGF), vascular endothelial growth factor-A (VEGF-A) and platelet-derived growth factor A (PDGF-A), that promote cell adhesion, angiogenesis and proliferation [28, 29]. During tumor metastases, it also induced epithelial to mesenchymal transition (EMT) by modulating the expression of miRNAs that promoted EMT-related gene expression [30, 31]. In this study, we suggested that MUC1 was highly expressed in recurrent LMS and LPS, suggesting a potential novel biomarker to predict STS recurrence. Additionally, as an extensively O-glycosylated and moderately N- glycosylated transmembrane protein on epithelial cells, many antibody-drug conjugates (ADC) have been explored for MUC1, such as HuHMFG1 [32, 33]. Thus, MUC1 can be regarded as a potential therapeutic target for recurrent STS.

Recently, MUC1 has also been designed to be the target of an anticancer vaccine. The MUC1 anticancer vaccine was equipped with covalently linked divalent mannose ligands and the mannose coupling also led to increasing numbers of macrophages, dendritic cells (DCs), and CD4^+^ T cells [34]. MUC1 could be carried in extracellular microvesicles, which played a contradictory role in promoting both immunosuppression and tumor growth. The specific immune response was reported to positively impact DCs immunogenicity by reprogramming DC antigen processing machinery and intracellular signaling pathways [35].

DCs, also known as professional antigen-presenting cells (APC), are specialized in providing co-stimulation and cytokines to regulate tumor antigen-specific T cell immune response activation [14]. They interact with other immune cells, such as NK cells and B cells, and activate anti-tumor responses [15]. The diversity of DC populations, divided by localization and activity, makes its function specific. The former includes Langerhans cells, monocyte-derived DCs (CD14^+^ DCs), myeloid DCs, plasmacytoid DCs (pDCs) [16]. The latter includes DCs activated and DCs resting [44].

In addition, both miR-1226 and MUC1 were reported to not only take effects in the intracellular environment but also be secreted to the extracellular environment which also provides the opportunity to regulate dendritic cells resting [35, 36].

In addition, correlation between hsa-let-7i-5p with dendritic cells resting was also significant (R = 0.200, P = 0.003). After a systematic literature review, we found no direct reports on hsa-let-7i-5p association with dendritic cells and tumor immunity. However, COP9 Signalosome Subunit 6 (COPS6) regulated by hsa-let-7i-5p had been proved by a previous study biological experiment [37]. COP9 Signalosome is a highly conserved protein complex, working as an important regulator in multiple signaling pathways. Especially, it had been reported to involve in the human immunodeficiency virus type 1 (HIV-1) regulating immune cell death [38–40]. Therefore, we speculated that hsa-let-7i-5p might affect dendritic cells by regulating its target gene COPS6, which was involved in immune regulation. We had such a conserved discussion because of the lack of literature. Subsequent studies are needed to explore the relationship between hsa-let-7i-5p and dendritic cells.

There are inevitably several limitations of our study that should be acknowledged. First, the amount of data released in publicly available datasets is limited, so that the clinicopathological parameters analyzed in this study are not comprehensive, which might lead to potential error or bias. And the sample size of the recurrent samples was very small, which might cause analysis bias. Second, we have not considered the heterogeneity of the immune microenvironment related to the location of immune infiltration. And the heterogeneity of the histological subtypes could affect the accuracy and generalization of the prediction models. Third, all data series downloaded for establishment of the prediction nomograms were from Western countries; thus, caution should be exerted when applying the conclusion of this study to patients from Asian countries. And to minimize bias, multiple databases were used to detect gene and protein expression levels of key biomarkers at the tissue and cellular levels, showing the key biomarkers were significantly upregulated in common sarcoma tissues and cell lines and their proteins were not expressed in normal soft tissues (Supplementary Figure 5–9). Last but not least, this study is only a correlation study on multiple dimensions rather than a biological mechanism study. However, notwithstanding its limitations, this study firstly established the nomograms to predict the survival of STS patients based on recurrent STS-specific tumor-infiltrating immune cells and ceRNA networks and inferred that the mechanism of hsa-miR-1226-3p regulating MUC1 and dendritic cells resting might play an important role in STS recurrence. In the future, more data should be incorporated to improve the model. As our future directions, we would investigate the direct molecular biological mechanisms of the recurrent STS-specific ceRNAs and the intercellular communication between cancer cells and dendritic cells resting.

## CONCLUSIONS

Our study constructed two nomograms to predict survival and recurrence of STS patients based on tumor-infiltrating immune cells and ceRNA networks, and demonstrated the utility by their high AUC values. The proposed prediction nomograms might provide much comprehensive clinical information for improving the personalized management of STS patients. Moreover, this study inferred that the mechanism of hsa-miR-1226-3p regulating MUC1 and dendritic cells resting might play an important role in STS recurrence.

## MATERIALS AND METHODS

### Data collection and differential gene expression analysis

The study was approved by the Ethics Committee of the First Affiliated Hospital of Zhengzhou University (No. 2019-KY-108). RNA profiles of the primary sarcomas and local recurrence samples were downloaded from TCGA (https://tcga-data.nci.nih.gov/tcga/) database. These samples were taken from patients confirmed as soft tissue sarcomas by histopathological diagnosis. All patients in this database have a uniform ID. The .Xml (Extensible Markup Language) files containing all the metadata for each sample was downloaded and merged by Practical Extraction and Report Language (Perl) script to determine the grouping of the samples in this study. Both HTseq-count and fragments per kilobase of exon per million reads mapped (FPKM) profiles of 262 samples, comprising 259 primary sarcomas and 3 local recurrence samples were collected (The specimens used for analysis in each experiment were primary/recurrent STS, not primary STS/normal tissue, or primary STS in patients with/without recurrence). Demographic information and survival endpoint of each patient were also retrieved.

After filtering non-sarcoma specific expression genes (No expression was detected in both experimental group and control group), the edgeR method was used to identify differentially expressed mRNAs, lncRNAs, and miRNAs. With a false discovery rate (FDR) P value < 0.05, the log(fold-change) > 1.0 or < -1.0 was defined a downregulated or upregulated gene, respectively.

### Construction of the ceRNA network

Before primary statistical analysis, the miRNA–mRNA interaction information based on experimental verification was download from miRTarBase (http:// mirtarbase.mbc.nctu.edu.tw/) [41] and while lncRNA–miRNA interaction information was download from lncbase v.2 Experimental Module (http://carolina.imis.athena-innovation.gr/diana_tools/web/index.php?r= lncbasev2%2Findex-experimental) [42]. Then, miRNAs regulated both lncRNAs and mRNAs showing significant results in hypergeometric testing and correlation analysis were selected for construction of the ceRNA network using Cytoscape v.3.5.1 [43].

### Survival analysis and nomograms of key members in the ceRNA network

Kaplan–Meier survival analysis and Cox proportional hazards model were generated to identify the prognostic value of all biomarkers. All significant biomarkers were integrated into the Cox model and the Lasso regression was performed to ensure that the multifactor models were not overfitting. Eventually, we built a nomogram based on the multivariable models to predict the prognosis of patients with sarcomas. The calibration curves and receiver operating characteristic curves (ROC) were utilized to assess the discrimination and accuracy of the nomogram.

### CIBERSORT estimation

In order to further explore the cytological causes of sarcoma-recurrence and molecular mechanism of the vital biomarkers in ceRNA network to some extent, the CIBERSORT algorithm [44] was used to estimate the fraction of 22 immune cell types in the primary and local recurrent sarcomas. Samples with a CIBERSORT output of P < 0.05 were considered to be eligible for further analysis. The Wilcoxon rank-sum test was implemented to find the immune cells, which had significant differences in the proportion between recurrent and primary tumors. Besides, Cox regression and Kaplan–Meier method were also applied to assess the relationship between the proportion of immune cells and sarcoma patients’ overall survival. Pearson correlation analysis was done for each prognostic biomarker in the ceRNA network and the proportion of each survival related immune cell. Finally, immune cells and biomarkers that were significantly associated with overall survival were incorporated into a nomogram.

### Immunohistochemistry (IHC)

Paraffin-embedded, formalin-fixed LMS and LPS specimens were used for IHC. Sections were incubated overnight in a humidified container at 4°C with the primary antibodies of MUC1 (1:100, ab109185; Abcam) and CD11c (1:100, ab52632, Abcam). After three times washing, tissue sections were incubated with the secondary antibody conjugated with streptavidin–horseradish peroxidases for 1 h at room temperature. The slides were stained with 3, 3-diaminobenzidine tetrahydrochloride (DAB) and the nuclei were counterstained with hematoxylin. Immunostaining on each slide was assessed by experienced pathologists to examine the percentage of MUC1 or CD11c positive tumor cells and presented as histochemistry score (H-score). H-score = Σpi(i+1) where i is the intensity score and pi is the percent of the cells with that intensity.

### Multidimensional validation

To minimize bias, multiple databases including the Gene Expression Omnibus (GEO) (ID: GSE21050 [45], GSE21122 [45], GSE6481 [46]. These three data sets were used for multidimensional external validation in the online database), Oncomine [47], Cancer Cell Line Encyclopedia (CCLE) [48], cBioPortal for Cancer Genomics [49, 50] Genotype-Tissue Expression (GTEx) [51], UCSC Treehouse Childhood Cancer Initiative [52], The Human Protein Atlas [53], CellMarker [55] were used to detect gene and protein expression levels of key biomarkers at the tissue and cellular levels.

### Statistical analysis

Only two-sided P value < 0.05 was thought to be statistical significance. All statistical analyses were enforced with R version 3.5.1 software (Institute for Statistics and Mathematics, Vienna, Austria; https://www.r-project.org) (Package: GDCRNATools [54], edgeR, ggplot2, rms, glmnet, preprocessCore, survminer, timeROC).

## Supplementary Material

Supplementary Figures

Supplementary Tables
