# Supplementary material for: The construction and analysis of tumor-infiltrating immune cell and ceRNA networks in recurrent soft tissue sarcoma
Source: Aging (Albany NY). 2019 Nov 18;11(22):10116–43. doi: 10.18632/aging.102424 (PMC6914407; doi:10.18632/aging.102424)
Supplement: Supplementary Figures [file aging-11-102424-s002..pdf]

SUPPLEMENTARY FIGURES

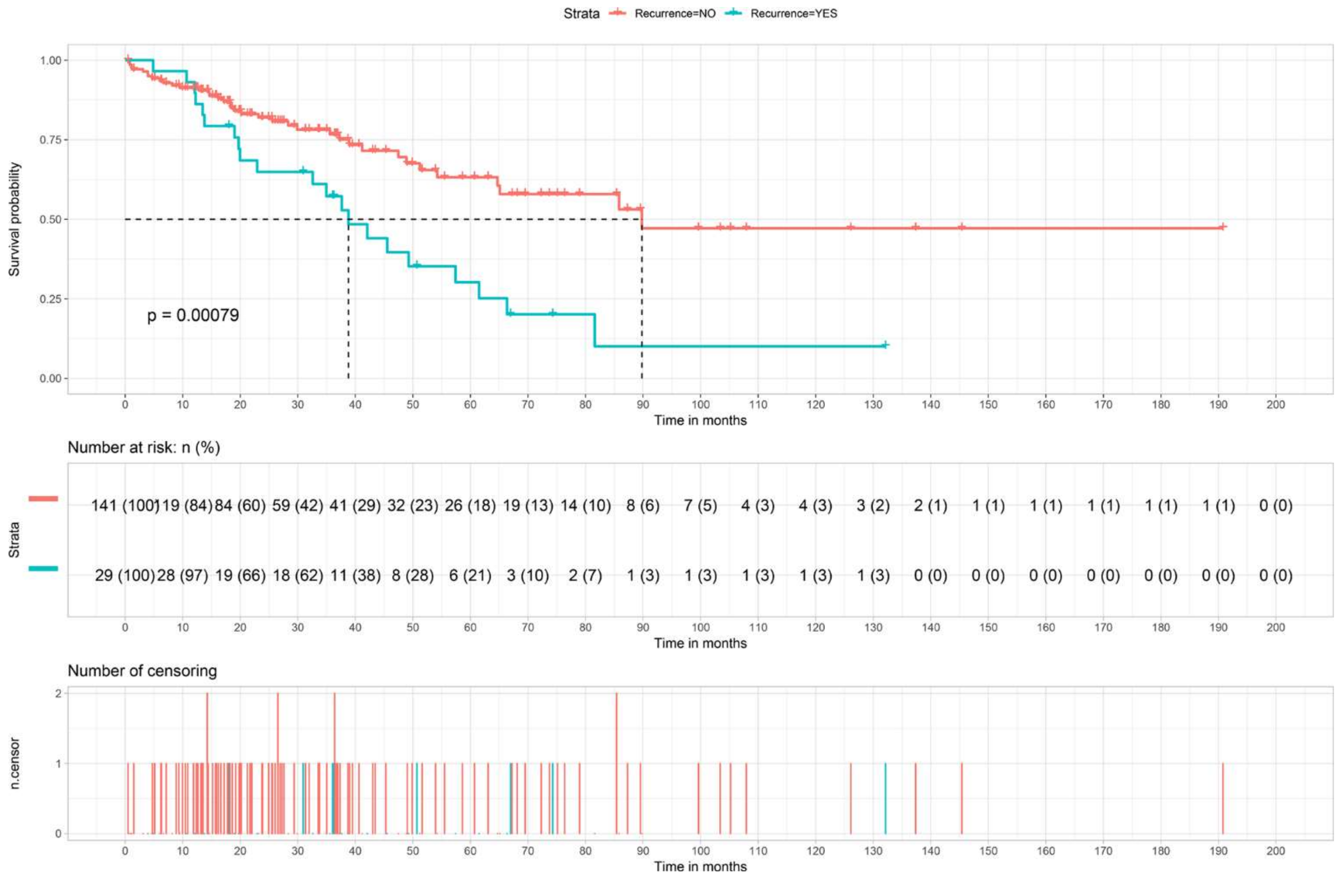

Supplementary Figure 1. **The Kaplan–Meier survival curve revealed that recurrence was a significant risk indicator for poor prognosis of STSs (P = 0.001).** Why is there a higher survival probability in first 10 months in those cases with STS recurrence? In general, solid tumor recurrence refers that the tumor reappears during the follow-up of patients after the primary operation. So, what this means is that patients who have had a recurrence of soft tissue sarcomas have had the primary operation. By reanalysis of the data, we found that most of the patients with survival time less than 10 months had tumor-bearing status (survive with tumor). Therefore, we speculated that these patients might have no chance to perform the primary operation, leading to poor prognosis. Tumor recurrence cannot happen to a patient who do not have the primary operation. Hence the phenomenon.

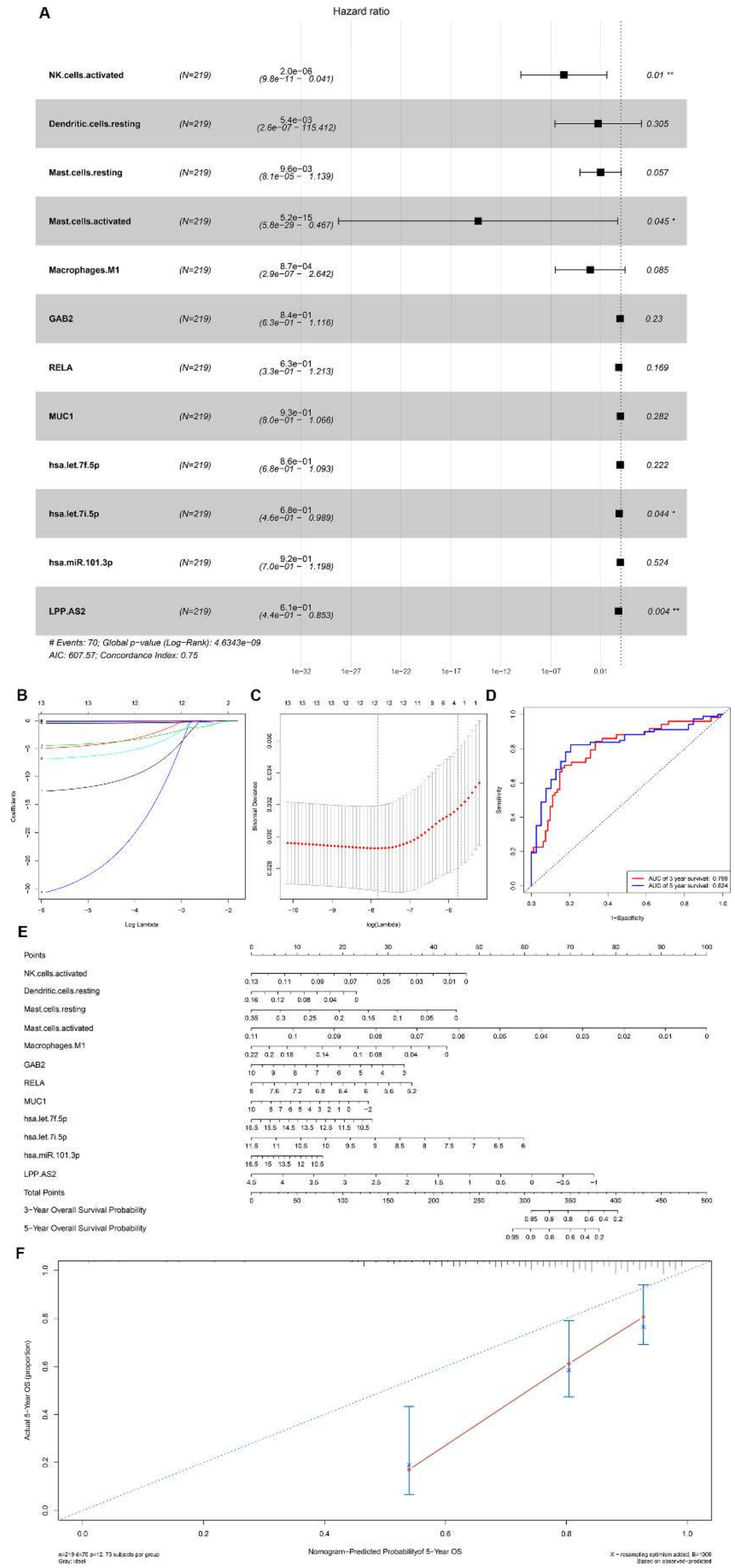
A
Hazard ratio
NK.cells.activated (N=219) 2.0e−06 (9.8e−11 − 0.041) 0.01 **
Dendritic.cells.resting (N=219) 5.4e−03 (2.6e−07 − 115.412) 0.305
Mast.cells.resting (N=219) 9.6e−03 (8.1e−05 − 1.139) 0.057
Mast.cells.activated (N=219) 5.2e−15 (5.8e−29 − 0.467) 0.045 *
Macrophages.M1 (N=219) 8.7e−04 (2.9e−07 − 2.642) 0.085
GAB2 (N=219) 8.4e−01 (6.3e−01 − 1.116) 0.23
RELA (N=219) 6.3e−01 (3.3e−01 − 1.213) 0.169
MUC1 (N=219) 9.3e−01 (8.0e−01 − 1.066) 0.282
hsa.let.7f.5p (N=219) 8.6e−01 (6.8e−01 − 1.093) 0.222
hsa.let.7i.5p (N=219) 6.8e−01 (4.6e−01 − 0.989) 0.044 *
hsa.miR.101.3p (N=219) 9.2e−01 (7.0e−01 − 1.198) 0.524
LPP.AS2 (N=219) 6.1e−01 (4.4e−01 − 0.853) 0.004 **
Events: 70; Global p−value (Log−Rank): 4.6343e−09
AIC: 607.57; Concordance Index: 0.75
1e−32 1e−27 1e−22 1e−17 1e−12 1e−07 0.01
B
Coefficients
Log Lambda
C
Binomial Deviance
log(Lambda)
D
Sensitivity
1−Specificity
AUC of 3 year survival: 0.799
AUC of 5 year survival: 0.824
E
Points
NK.cells.activated
Dendritic.cells.resting
Mast.cells.resting
Mast.cells.activated
Macrophages.M1
GAB2
RELA
MUC1
hsa.let.7f.5p
hsa.let.7i.5p
hsa.miR.101.3p
LPP.AS2
Total Points
3−Year Overall Survival Probability
5−Year Overall Survival Probability
F
Actual 5−Year OS (proportion)
Nomogram−Predicted Probabilityof 5−Year OS
n=219 d=70 p=12, 73 subjects per group
Gray: ideal
X − resampling optimism added, B=1000
Based on observed−predicted

Supplementary Figure 2. **The results of multivariable model and the nomogram integrating immune cells and biomarkers significantly associated with overall survival.** Key members of the ceRNA network and immune cells were integrated into one Cox regression model (**A**) After the screening process of the Lasso regression, the results suggested that the model was not overfitting (**B**, **C**). The calibration curve and the ROC demonstrated good discrimination and concordance of the multivariable model (AUC of 3-year survival: 0.799; AUC of 5-year survival: 0.824) (**D**, **F**). The nomogram was constructed based on the multivariable model (**E**).

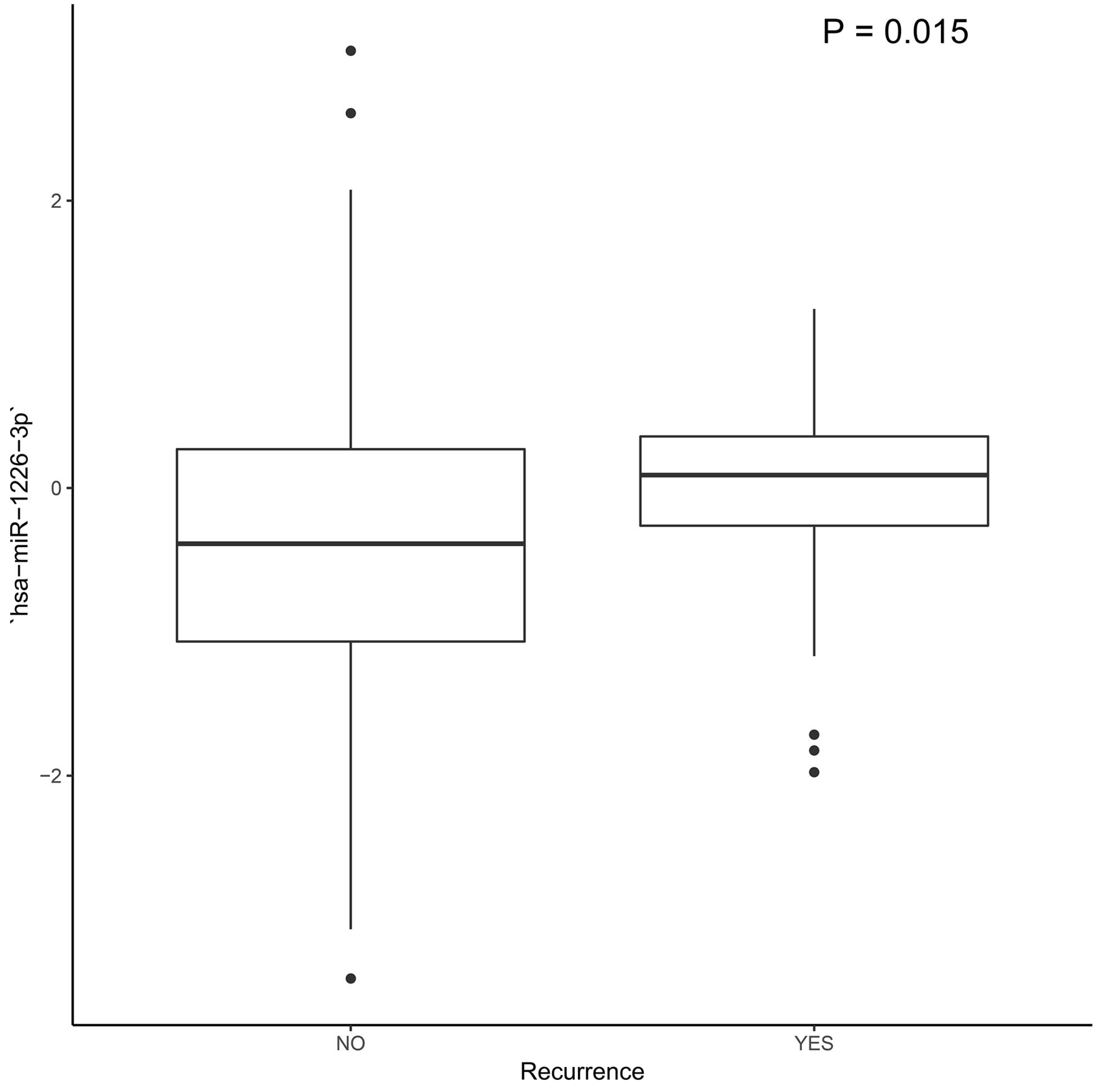

Supplementary Figure 3. **The result of the Wilcoxon rank-sum test suggesting that the hsa-miR-1226-3p expression level is significant differences between the primary sarcoma tissues of patients with and without recurrence (P = 0.015).**

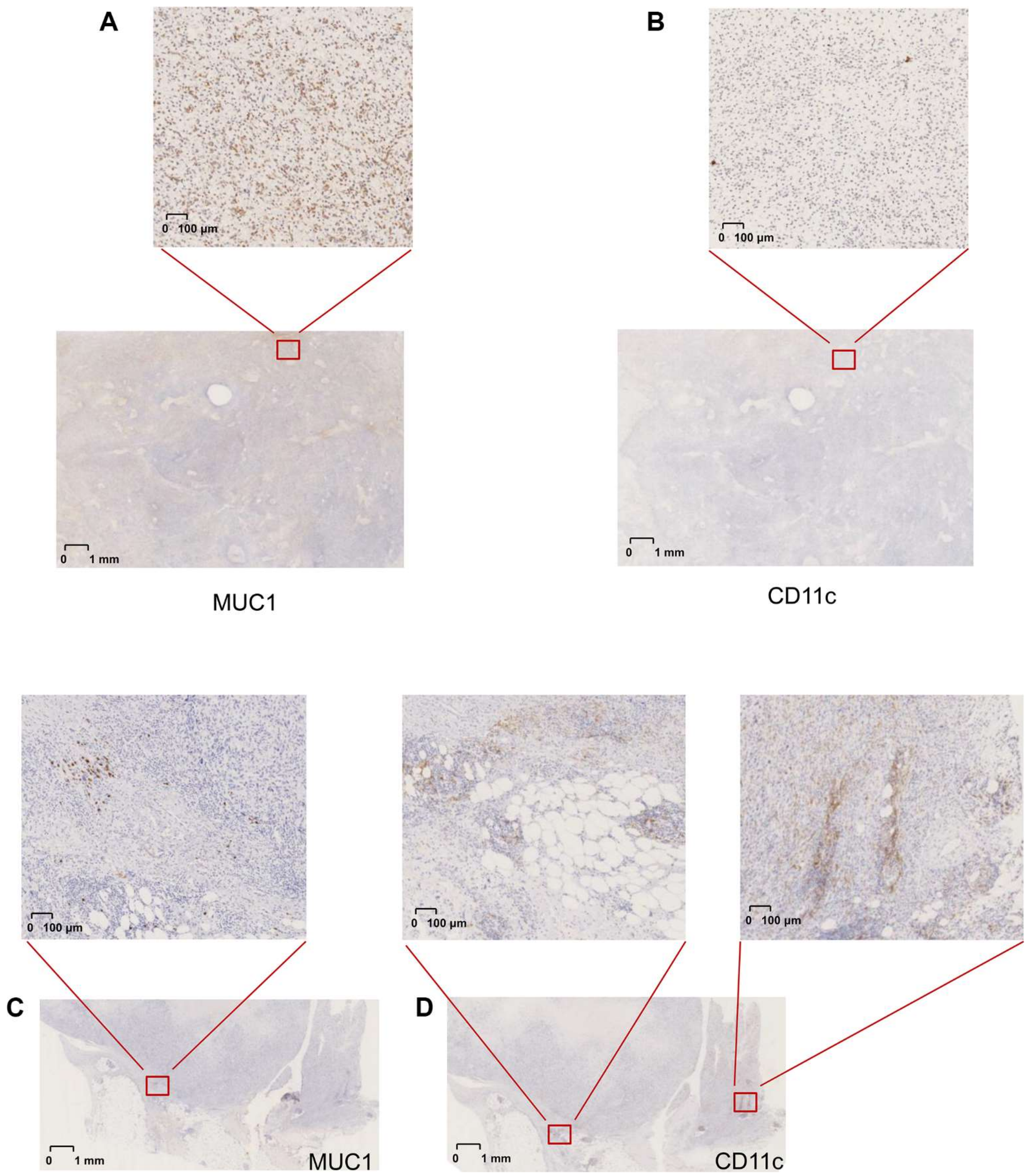

Supplementary Figure 4. **The results of the immunohistochemistry (IHC) stain showing that MUC1 and CD11c were correlated with STS recurrence and predominantly localized in the membrane and extracellular matrix of leiomyosarcoma and liposarcoma cells.** MUC1 (**A**) and CD11c (**B**) protein was predominantly localized in the membrane and extracellular matrix of LMS. MUC1 (**C**) and CD11c (**D**) protein was predominantly localized in the membrane and extracellular matrix of LPS. Abbreviations: LMS: leiomyosarcoma; LPS: liposarcoma.

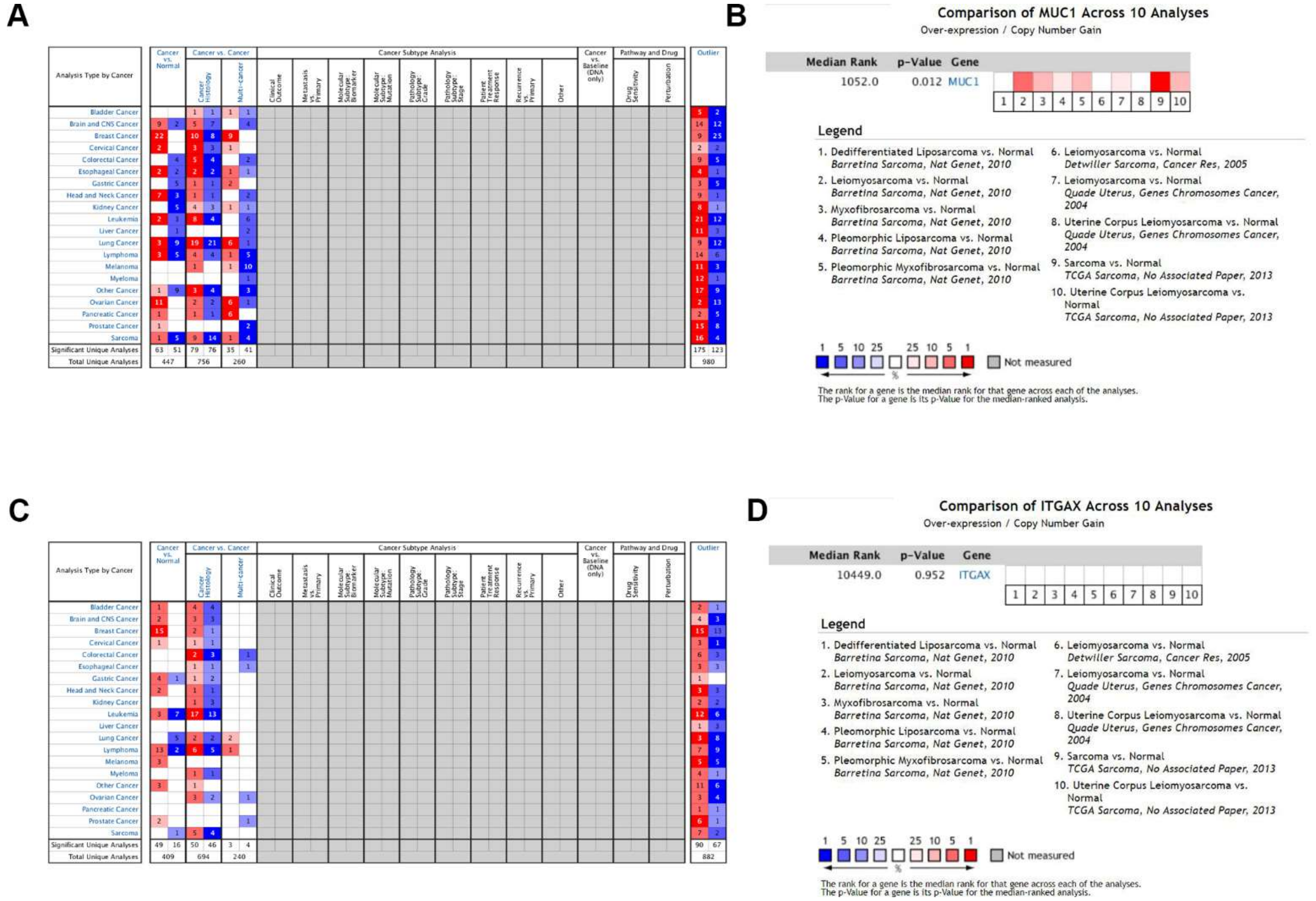

Supplementary Figure 5. Validation of MUC1 (**A**, **B**) and CD11c (**C**, **D**) on a transcriptional level in multiple cancer types and multiple studies using the Oncomine database.

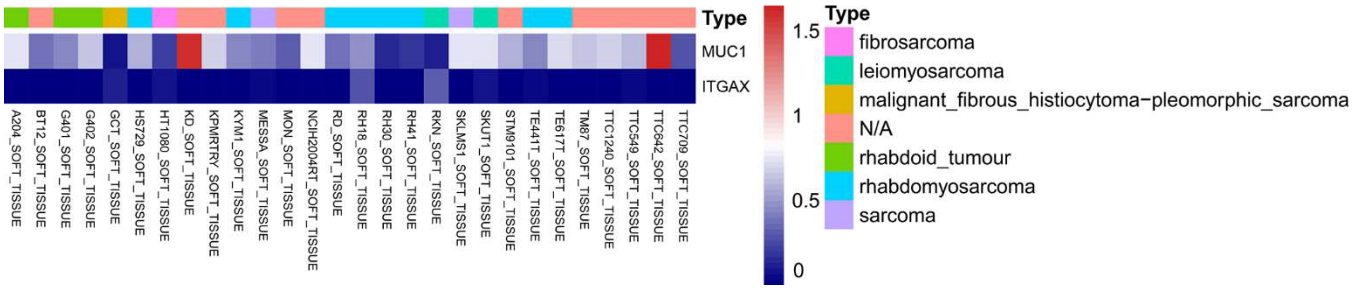

Supplementary Figure 6. The expression levels of MUC1 and CD11c in various soft tumor cell lines in Cancer Cell Line Encyclopedia (CCLE).

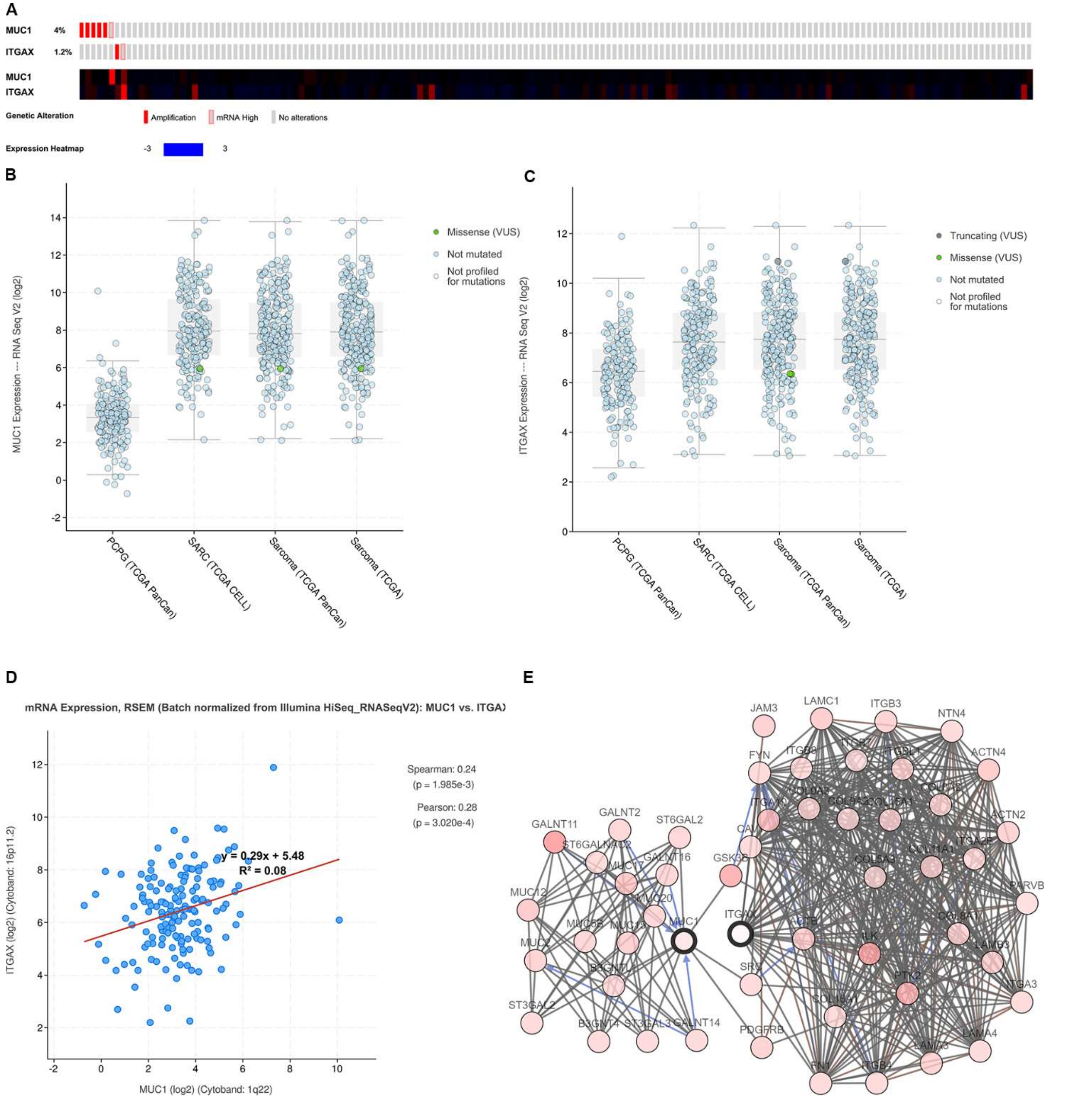

Supplementary Figure 7. **Integrative analysis of genomics and clinical profiles using the cBioPortal database.** (**A**) Alteration frequency of MUC1 and CD11c; (**B**, **C**) MUC1 and CD11c were highly expressed in primary STS compared to some other types of cancer; (**D**) The co-expression between MUC1 and CD11c. (**E**) The Protein-Protein Interaction (PPI) network of MUC1 and CD11c.

A

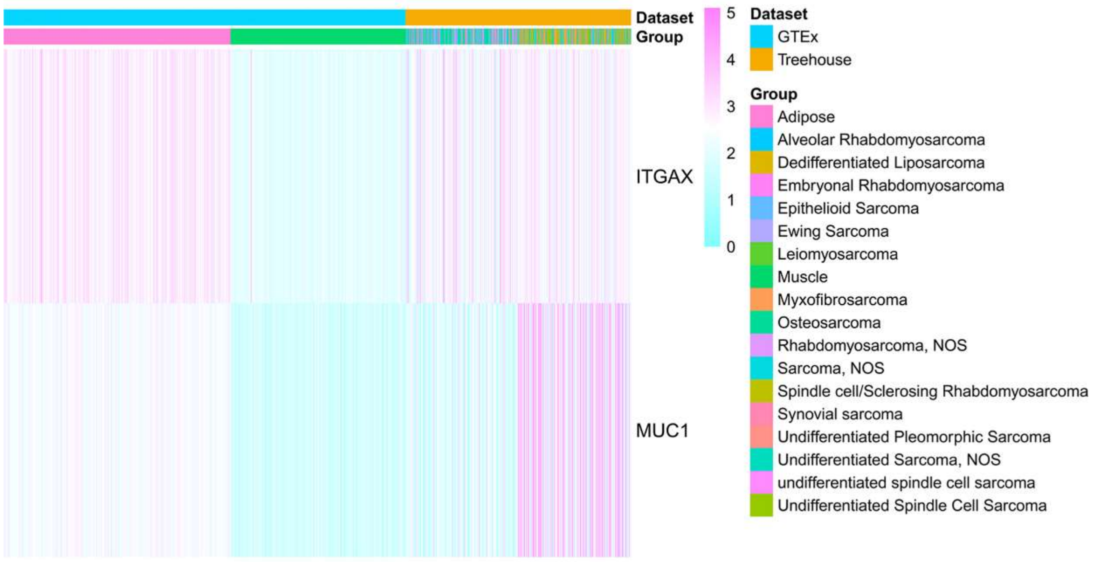

B

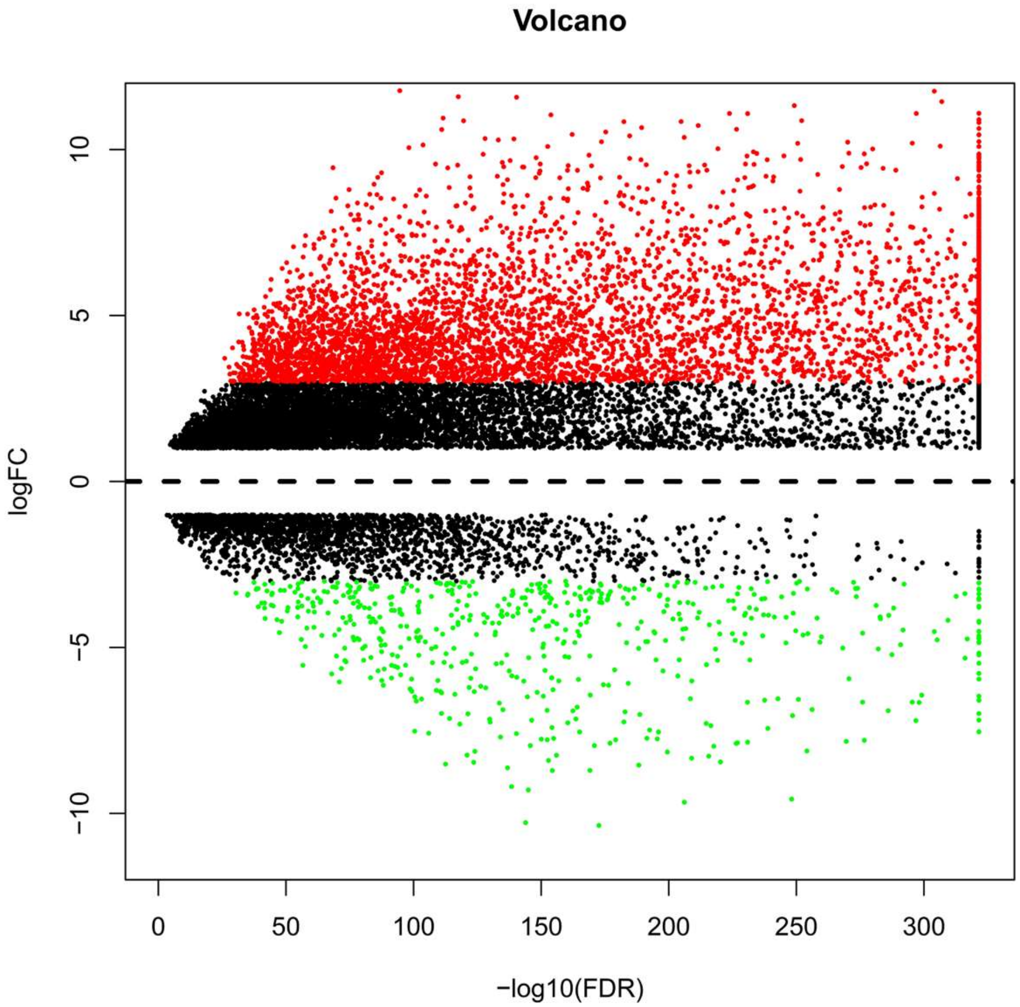

Supplementary Figure 8. Differential gene analysis heatmap of (**A**) MUC1 and CD11c and volcano plot (**B**) using data from the GTEx database and the Treehouse database.

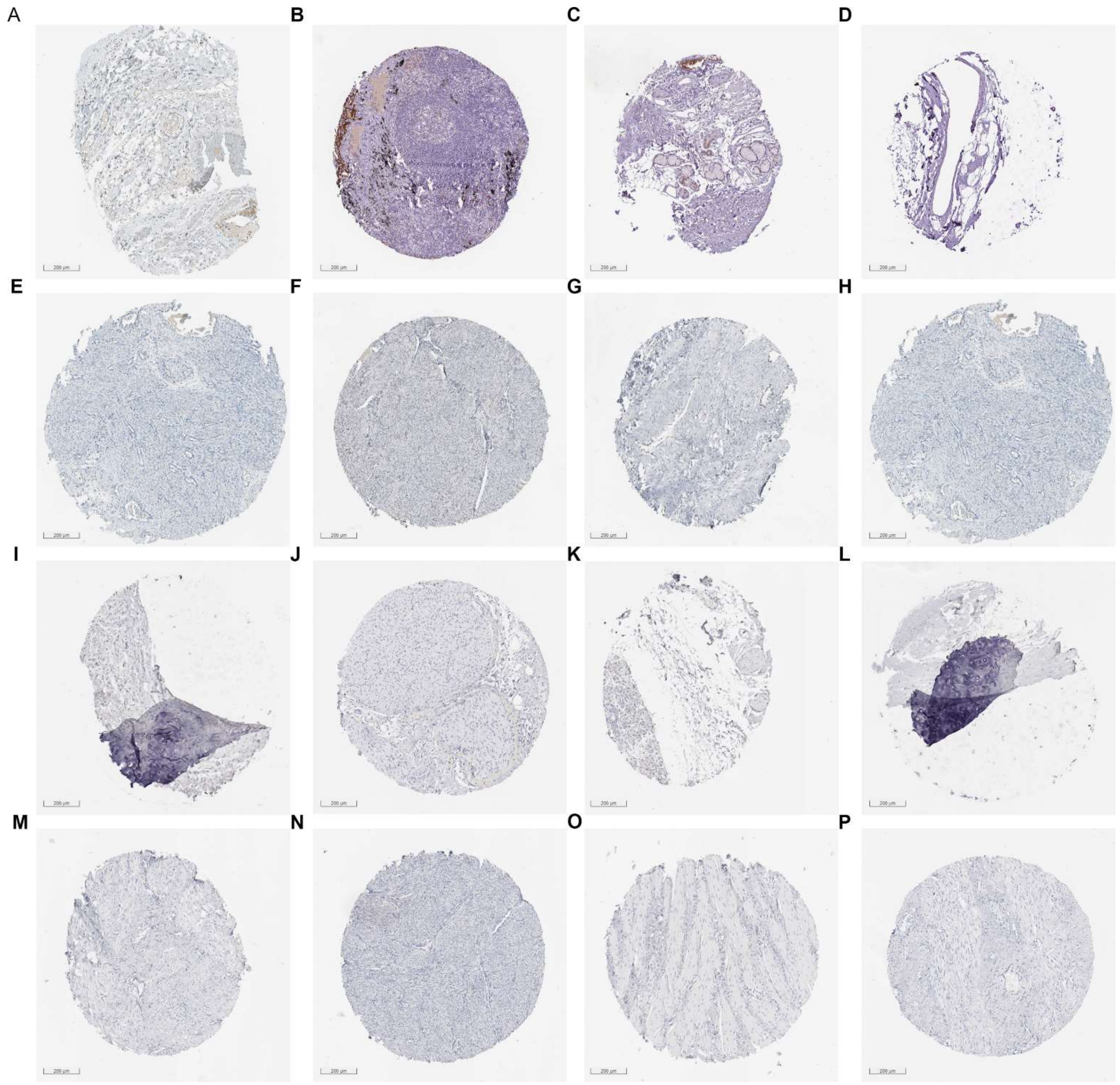

Supplementary Figure 9. Validation of MUC1 (Adipose: **A**–**D**; Smooth muscle: **E**–**H**) and CD11c (Adipose: **I**–**L**; Smooth muscle: **M**–**P**) on a translational level using the Human Protein Atlas database. The results of data mining of The Human Protein Atlas showed that the protein MUC1 and CD11c were almost not detected in normal adipose tissue and smooth muscle tissue.

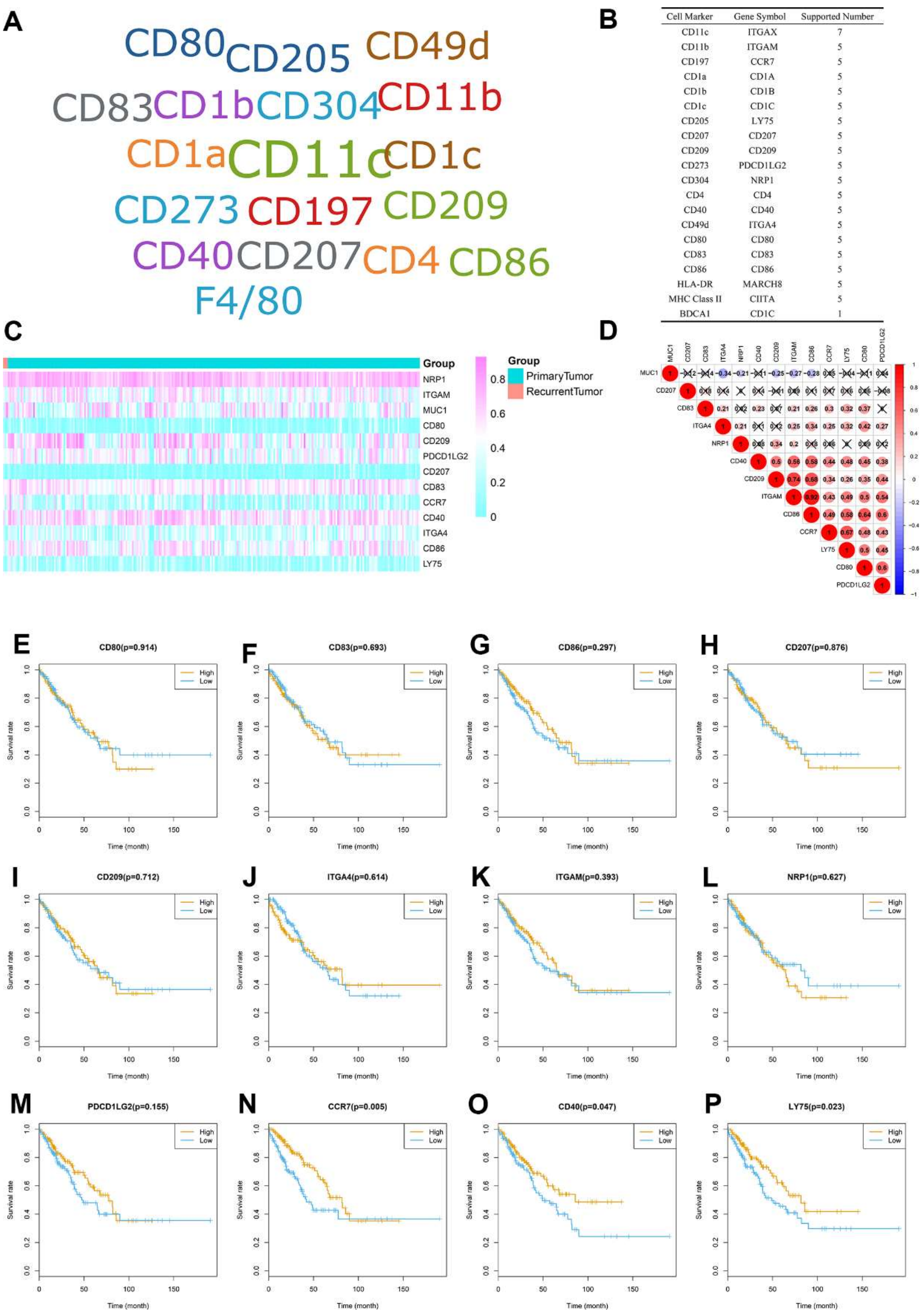

Supplementary Figure 10. **Evaluation prognostic value and relationship with MUC1 of all specific surface markers of dendritic cell.** On the basis of detecting CD11c, we determined all surface markers of dendritic cell reported for more than 5 times in previous studies by CellMarker database. After removing nonspecific surface markers, CD11b, CD197, CD205, CD207, CD209, CD273, CD304, CD40, CD49d, CD80, CD83, CD86 were integrated into further validation. (**A**, **B**) All surface markers of dendritic cell in CellMarker database; (**C**) Heatmap of twelve markers and MUC1; (**D**) Co-expression heatmap of twelve markers and MUC1; (**E**–**P**) Kaplan–Meier survival curves of twelve markers.
