# Supplementary material for: The construction and analysis of tumor-infiltrating immune cell and ceRNA networks in recurrent soft tissue sarcoma
Source: Aging (Albany NY). 2019 Nov 18;11(22):10116–43. doi: 10.18632/aging.102424 (PMC6914407; doi:10.18632/aging.102424)
Supplement: Supplementary Tables [file aging-11-102424-s001..pdf]

SUPPLEMENTARY TABLES

Supplementary Table 1. Baseline information of 261 patients diagnosed with soft tissue sarcoma.

Variables	Total Patients (N = 261)
**Age, years**	
Mean ± SD	60.87 ± 14.62
**Gender**	
Female	142 (54.41%)
Male	119 (45.59%)
**Race**	
Asian	6 (2.30%)
Black or African American	18 (6.90%)
White	228 (87.36%)
Unknown	9 (3.44%)
**Histological subtype**	
Dedifferentiated Liposarcoma	59 (22.61%)
Leiomyosarcoma	105 (40.23%)
Undifferentiated Pleomorphic Sarcoma	21 (8.05%)
Malignant Peripheral Nerve Sheath Tumors	9 (3.44%)
Myxofibrosarcoma	25 (9.58%)
Undifferentiated Pleomorphic Sarcoma	29 (11.11%)
Synovial Sarcoma	10 (3.83%)
Desmoid Tumor	2 (0.77%)
Undifferentiated Pleomorphic Sarcoma With Giant Cells	1 (0.38%)
**Recurrence**	
Yes	29 (11.11%)
No	144 (55.17%)
Unknown	88 (33.72%)

Abbreviations: SD: Standard deviation.

Supplementary Table 2. The list of top 10 downregulated and top 10 upregulated genes in differential gene analysis.

Gene	Type	LogFC	P value	FDR
CEND1	protein coding	-4.28656	2.09E-07	0.000116
CHRDL2	protein coding	-4.09075	7.90E-05	0.010565
PGR	protein coding	-4.0102	5.88E-05	0.008329
S100A3	protein coding	-3.96647	9.09E-11	1.88E-07
NT5M	protein coding	-3.96283	2.10E-14	1.52E-10
PODXL	protein coding	-3.90095	1.32E-20	1.91E-16
ESR1	protein coding	-3.60849	1.85E-05	0.003478
CAMK2N2	protein coding	-3.47569	1.73E-07	0.000104
AC105277.1	long non coding	-3.4071	2.01E-09	2.90E-06
RHOBTB3	protein coding	3.825303	0.000402	0.035656
PDGFRB	protein coding	3.196464	0.000375	0.034536
MTSS1	protein coding	2.728508	0.00025	0.025802
HMGN1	protein coding	1.728704	0.000592	0.048322
SNX14	protein coding	1.340009	8.38E-06	0.001893
RELA	protein coding	-0.78947	0.000562	0.046364
ARMC10	protein coding	-0.94507	0.000287	0.028824
PDCD7	protein coding	-0.95058	0.000204	0.022307
ZNF3	protein coding	-0.96601	0.000162	0.018492
PMPCB	protein coding	-0.96741	2.15E-05	0.003875

Abbreviations: FC: Fold change; FDR: False Discovery Rate.
